# Learning effects of different training models for border molding from the perspective of dental students

**DOI:** 10.1186/s12903-017-0443-9

**Published:** 2017-12-16

**Authors:** Mai Okubo, Yusuke Sato, Yuki Hirajima, Shunsuke Minakuchi

**Affiliations:** Department of Gerontology and Gerodontology, Graduate School, Gerodontology and Oral Rehabilitation, Tokyo Medical and Dental University, 1-5-45 Yushima, Bunkyo-ku, Tokyo, 113-8549 Japan

**Keywords:** Border molding, Dentures, Practical training, Undergraduate student

## Abstract

**Background:**

The aim of this study was to investigate the effects of different practical training models on the comprehension and evaluation of practical training among dental students.

**Methods:**

The study subjects were all sixth-year dental students at our institute, and the study took place over three consecutive years (*n* = 58, 63, and 65, respectively). In practical training, all students learned border molding, and practical models were modified each year from plaster models to silicone models and then to silicone models mounted in mannequins. Immediately after completing clinical training, all students were asked to complete questionnaires consisting of 21 items regarding their overall practical training and their clinical comprehension of border molding. All items were rated on a five-point Likert scale, and in order to reduce the large number of interrelated questions, exploratory factor analysis was carried out using maximum likelihood estimation with promax rotation (κ = 4) and Kaiser normalization. The number of factors was chosen using the Kaiser-Guttman rule, which states that the eigenvalue should be larger than 1, and the scree plot criteria. Items that scored less than 0.25 in communality and exhibited factor loading greater than 0.35 for more than one item were excluded. The defined factors were analyzed for the plaster models, the silicone models alone, and the silicone models with mannequins using the Kruskal-Wallis test and follow-up tests using Bonferroni-corrected Mann-Whitney U tests. The significance level was set at *p* < 0.05.

**Results:**

Exploratory factor analysis identified the following three factors: “knowledge of border molding”; “contents of practical training”; and “personal learning attitude”. The students who used silicone models and mannequins gave significantly better evaluations on the “knowledge of border molding” (*p* < 0.001, both) and “contents of practical training” (*p* = 0.046, *p* < 0.001, respectively) subscales than those who used plaster models. No significant differences were observed between those who used silicone models and those who used mannequins. Moreover, no significant differences were found on the “personal learning attitude” subscale among students for any model.

**Conclusions:**

The change in practical training models from plaster to silicone improved student evaluations of border molding training.

**Electronic supplementary material:**

The online version of this article (10.1186/s12903-017-0443-9) contains supplementary material, which is available to authorized users.

## Background

Although Japan has rapidly become a super-aged society, with more than one-fourth (26.8%) of the total population aged 65 years or older [1], a survey of dental diseases conducted in Japan in 2011 reported a decrease in the number of edentulous patients and complete denture wearers [2]. While the results of the next survey will not be reported until 2017, this trend is expected to continue. This decrease has also been observed in other industrialized countries, and now that treatment for edentulous patients requires higher levels of expertise and technical skills than ever before, it is increasingly difficult for general dentists to fabricate complete dentures [3].

One of the most important steps of denture fabrication is impression taking. Boucher reported that the primary objectives of complete denture impressions were as follows: 1) retention; 2) stability; 3) support; 4) esthetic value; and 5) preservation of the alveolar ridge [4]. In other words, impression taking is an integral component of complete dentures. Therefore, the accuracy of the impression greatly affects the quality of the dentures and the level of patient satisfaction [5, 6]. Although complete dentures have been described as having a fundamental form based on anatomy [7], a substantial part of the complete denture border is due to its location in the area of the mucobuccal fold. In the case of mandibular dentures, the border must be located beyond the point of muscle attachment, which complicates the border molding procedure for general dentists [8]. Dental compounds are useful for defining this unclear border, but dentists must learn this technique through practical experience, and the opportunities to do so are limited.

The dental curriculum for complete denture prosthodontics at Tokyo Medical and Dental University (TMDU) consists of lectures and practical training in the fourth year and clinical training in the fifth and sixth years. As part of practical training, students practice border molding in the practice room. Previously, students used plaster models to pattern impression material (Impression Compound, Kerr Corp., Orange, CA, USA) in custom impression trays. Students learning border molding should replicate clinical conditions as closely as possible during practical training in order to improve their skills [9]; however, the operation of the plaster model in the practice room is quite different from that actually experienced in the clinical setting. Therefore, the objective of this study was to gain familiarity with the actual materials used in border molding, not the technique itself.

Silicone models (G10-X1231A, Nissin Dental Products Inc., Kyoto, Japan) have been used in practical training since 2009. Silicone models have elastic qualities, high edges, and simulated tongues, and they are therefore more similar to the oral mucosa than plaster models. In 2010, all dental students were provided with mannequins (Phantom DR-11, J. Morita Mfg. Corp., Kyoto, Japan) in which silicone models could be placed; this allowed better replication of clinical conditions than working with a silicone model alone.

In order to promote further improvements in training content and enhance learning efficiency, the learning effects of silicone training models for border molding were investigated from the perspective of dental students.

## Methods

### Summary of practical training

Border molding training for mandibular dentures is part of the practical training carried out in the fourth year for dental students at TMDU. During training, each student prepares a custom impression tray made of autopolymerizing acrylic resin. Through 2008, students first applied petroleum jelly on the surface of plaster models, heated the impression materials onto the border of the trays, placed them briefly in warm water, and then placed the trays on the models. Thereafter, the students used their fingers to apply direct pressure to the compound and mold the model form.

After silicone models were introduced in 2009, students in practical training added the compound to the trays and placed them on the models, similar to the technique used for plaster models. They then applied pressure to the edges of the silicone models. The silicone models have artificial tongues and longer edges, which allow students to apply varying degrees of pressure to bend the model in the manner required.

**Fig. 1:**
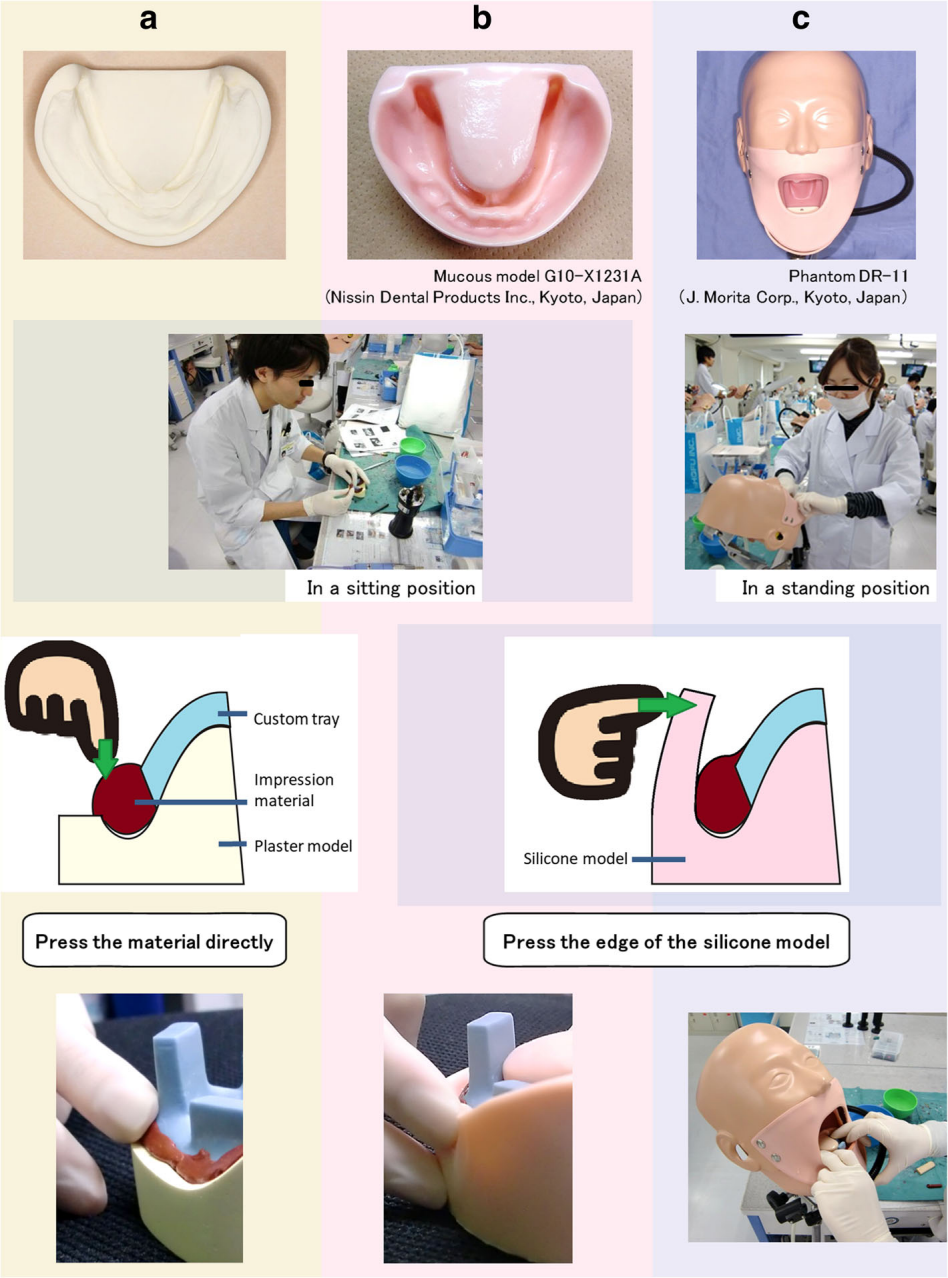
Border molding training schema: (**a**) plaster model, press the compound directly with fingers; (**b**) silicone model, press the edges of the silicone model; and (**c**) mannequin with silicone model, use in the same manner as the silicone model

In 2010, the silicone models were attached to the mannequins, but the technique used to mold the borders remained the same. However, the mannequins had openings for the mouth, a limited handling direction, and an adjustable height and head angle, which allowed students to mold the borders in a standing position, which is more consistent with actual clinical conditions. Students were given guidance by instructors throughout the training, and border molding was considered to be complete when the custom trays were completely filled with material (Fig. 1).

### Questionnaire protocol

The participants in this study were sixth-year dental students between 2010 and 2012. The practical models were plaster models in 2010, silicone models in 2011, and silicone models mounted in mannequins in 2012. All students were asked to complete an anonymous questionnaire immediately after completion of their clinical training. The questionnaire was composed of 21 items: 10 of these items related to their clinical comprehension of border molding, and the remaining 11 related to evaluations of the overall practical training. All items were rated on a five-point Likert scale (Additional file 1).

This study was carried out with the approval of the Ethics Committee of the Faculty of Dentistry at TMDU (registration number: 915) following their review of the questionnaire; the Committee confirmed that the results could be used without the students’ consent.

### Analysis

Students rated all items related to their comprehension of border molding and their level of satisfaction with the overall practical training on a five-point Likert scale as follows: 5 = “yes, definitely”; 4 = “I think so”; 3 = “no opinion”; 2 = “I don’t think so”; and 1 = “no, definitely not”. The mean scores for each year were then calculated.

In order to reduce the large number of interrelated questions to a smaller number of underlying common factors, exploratory factor analysis was carried out using maximum likelihood estimation with promax rotation (κ = 4) and Kaiser normalization. The number of factors was chosen using the Kaiser-Guttman rule, which states that the eigenvalue should be larger than 1, and the scree plot criteria. Items that scored less than 0.25 in communality and exhibited factor loading greater than 0.35 for more than one item were excluded.

The internal consistency of the subscales was assessed using Cronbach’s alpha coefficient. The defined factors were analyzed for the plaster models, the silicone models alone, and the silicone models with mannequins using the Kruskal-Wallis test and follow-up tests using Bonferroni-corrected Mann-Whitney U tests. The significance level was set at *p* < 0.05. All statistical analyses were performed using SPSS software (version 8.0; IBM SPSS PASW Statistics Base 17.0; IBM Japan, Tokyo, Japan).

## Results

The response rates were 48.3% (28 students) with plaster models, 82.5% (52 students) with silicone models, and 92.3% (60 students) with mannequins (Table 1), and the questionnaire results were shown in Additional file 2.

**Table 1:** Number of students and mean scores for the questionnaire items

	Responses/total (response rate)	Mean score
Plaster model	28/58 (48.3%)	3.573
Silicone model	52/63 (82.5%)	3.941
Mannequin	60/65 (92.3%)	4.017

As shown in Table 2, exploratory factor analysis identified three factors. Five questions (“Did you understand the technique for border molding on the labial side?”; “Did you understand the anatomic locations?”; “Are important matters adequately emphasized?”; “Did you understand the technical steps?”; and “Were you motivated for clinical training?”) were excluded from analysis because of insufficient factor loading. The Kaiser-Meyer-Olkin measure was 0.846, and Bartlett’s test of sphericity showed a significant difference between variables (*p* < 0.001). These results validated the application of exploratory factor analysis. In addition, the validity of the three factors was confirmed by the Kaiser-Guttman rule and the scree plot criteria.

**Table 2:** Results of exploratory factor analysis with each item divided into three factors

		Factor		
	Items	1	2	3
5.	Did you understand the method for border molding at the buccal side?	.957	.029	−.087
7.	Did you understand the method for border molding at the lingual side?	.953	−.095	.010
4.	Did you understand the method for border molding at the retromolar pad?	.821	.077	−.053
3.	Did you understand how to adjust the custom trays?	.649	.113	−.039
9.	Did you understand the mobility of mucosa?	.643	−.292	.134
1.	Did you understand how to use the impression material?	.588	.061	−.080
8.	Did you understand the image of the denture shapes?	.449	.058	.231
2.	Did you understand how to use the alcohol torch?	.446	.129	.117
19.	Is practical training well organized?	−.113	.794	−.052
13.	Did you understand the instructions of the teacher?	.083	.738	−.094
21.	Do you value practical training overall?	.161	.645	.046
20.	Is practical training easily comprehensible?	−.004	.606	.106
12.	Was the level of practical training appropriate for students?	−.059	.538	.140
15.	Did you find proper literature and references?	.061	−.081	.840
14.	Did you prepare for or review practical training?	−.027	.032	.785
16.	Are you satisfied with what you have learned?	−.046	.168	.652
	Interfactor correlation			
	1	–	.339	.459
	2		–	.449
	3			–
	Percent of variance explained	33.309	13.669	6.813
	Cronbach’s alpha	.893	.800	.818
	Mean	3.700	4.390	3.380
	SD	.991	.748	1.040

*SD* standard deviation

The first factor, consisting of eight items, showed high factor loading for items specific to the molding technique, such as “Did you understand the method for border molding on the buccal side?”. Therefore, this was termed the “knowledge of border molding” factor. The second factor, consisting of five items, showed high factor loading for items specific to the practical environment, such as “Is practical training well-organized?”. Therefore, this was termed the “contents of practical training” factor. The third factor, consisting of three items, showed high factor loading for items specific to the students’ attitudes, such as “Did you find proper literature or references?”, and was therefore termed the “personal learning attitude” factor.

As shown in Table 2, a weak positive correlation was observed between “knowledge of border molding” and “contents of practical training”. Medium positive correlations were observed between “knowledge of border molding” and “personal learning attitude”, and between “contents of practical training” and “personal learning attitude”. The internal consistencies of the three subscales were good because Cronbach’s alpha coefficients were greater than 0.8.

When comparing the three student groups on the “knowledge of border molding” subscales, the students using silicone models and those using mannequins provided significantly better evaluations than those using plaster models (*p* < 0.001, both), and on the “contents of practical training” subscales, the students using silicone models and those using mannequins provided significantly better evaluations than those using plaster models (*p* = 0.046, *p* < 0.001, respectively). However, no significant differences were observed between those using silicone models and those using mannequins. In addition, no significant differences were found among the three groups on the “personal learning attitude” subscale (Table 3).

**Table 3:** Results of multiple comparisons with each factor analyzed for the three model types

	Plaster model	Silicone model	Mannequin
Knowledge of border molding	3.18 ± 1.14^b^	3.84 ± 0.98^a^	3.83 ± 0.83^a^
Contents of practical training	4.19 ± 0.80^b^	4.38 ± 0.74^a^	4.50 ± 0.71^a^
Personal learning attitude	3.26 ± 1.02^a^	3.28 ± 1.13^a^	3.53 ± 0.95^a^

Data are expressed as means ± standard deviation. Different lowercase letters indicate significant differences among three model types considering each line

## Discussion

Although the current method for border molding is very popular, the practical environment varies among countries and dental schools. According to the previous studies in the United Kingdom [3, 10, 11], students have limited opportunities to fabricate complete dentures due to decreasing lecture times and declining numbers of edentulous patients. Instead, they are forced to make copy or repair dentures [12, 13]. Although this is also required knowledge, it is not fundamental knowledge. In the United States, many dental schools and postgraduate curricula continue to use custom tray impressions with silicone after border molding with dental compound [14–16]. While some studies suggest that using dental compounds requires advanced skills, others suggest that adequate training can be achieved through the treatment of several cases [9, 16]. It is true that patients exhibit different denture shapes and muscle activation patterns, and dentists should familiarize themselves with these differences through clinical experience. However, as was mentioned by Swenson [7], complete dentures do have a basic form, and skills cannot be applied without mastery of the fundamentals. As the number of patients continues to decline, practice with models can provide a proper foundation for students’ clinical skills.

Many studies have investigated the effect of denture impression method. As discussed in a review [17], a few studies with high-quality evidence indicated that the simplified impression method (single alginate impression) showed better results than the conventional impression method (preliminary and final impression), primarily in cost and time [18–22]. However, the high-quality papers such as those mentioned above came from only four research groups. There are a few who advocate the necessity of the two-step impression method [23]. Whichever method is chosen, additional research is needed. In many countries, dentists take impressions using border molding, which alludes to the fact that border molding is valuable for the treatment of edentulous patients.

This study did have some limitations. The response rate for plaster models was quite low, at 48.3%, and it was likely influenced by the time of collection. In addition, the reliability of the results is low, since the questionnaire was only conducted with students from one dental school. However, the histograms of the three student groups showed the same frequency distribution and had the same median with the highest frequency; therefore, the samples were considered valid for statistical analysis. However, the results of this study should not be over-generalized, since high external validity is not likely with just one study. Therefore, the external validity of this field will increase as many researchers complement each other.

The three factors extracted in this study were matched with the factors in the planning phase. Cronbach’s alpha coefficients were greater than 0.8, indicating the high credibility of the results. Moreover, intermediate correlations were observed between two of the three factors, indicating the adequacy of the exploratory factor analysis.

Students’ self-evaluations for “knowledge of border molding” were higher for silicone than for plaster models, and for mannequins than for plaster models. In silicone models, the edges are pressed to form the border indirectly, whereas in mannequins, the structures on the model resembling the lips or buccal mucosa hinder border molding. However, this is not the case with plaster models. Therefore, silicone models and mannequins are more suitable than plaster models for border molding practice. Furthermore, the change from silicone models to mannequins did not affect the students’ self-evaluations, indicating that the limitations created by mannequins did not have a drastic effect on evaluations compared with the change from plaster to silicone models. In addition, the evaluations for the silicone models and mannequins might have been too high to allow any differences to be seen.

The “contents of practical training” was also significantly affected by the change in models, especially from plaster models to mannequins. Many dental schools in the Unites States have used mannequins for more than 10 years, but their specifications and the effects of their use remain unclear [24]. Mannequins have been used to take 73.7% and 57.9% of all primary and final impressions, respectively, in Spain and Portugal [25]. It therefore seems that the use of mannequins has not achieved a high degree of legitimacy. Mannequins are effective for bridging the gap between the laboratory and clinical setting, which, in addition to the learning effects, helps students develop a clearer understanding of actual treatment procedures. The use of different models might contribute to an improvement in the overall evaluation of practical training.

No significant differences were observed in “personal learning attitude” in this study, but mean scores were highest with the mannequin, followed by the silicone and plaster models in descending order. This result indicates the possibility that practice with models had a positive effect. Each student worked with and assessed only one training model, and this should be kept in mind when considering the validity of the results.

The purpose of using silicone models is to help students comprehend the characteristics of modeling compounds and develop skills in border molding. Silicone models are already used in postgraduate training courses and objective structured clinical examinations. In the future, it is expected to expand the use of the silicone models for general improvement in treatment, and border molding training should include movable and immovable mucosa and free lingual movement to make it more consistent with actual clinical conditions. Considering the limited time and number of cases included in the dental curriculum, silicone models are expected to allow an adequate mimicking of clinical conditions and to foster better impression-taking skills.

## Conclusions

Within the limitations of this study, changing the practical training models from plaster to silicone appeared to improve student comprehension and evaluation of practical training.

## Additional files

Additional file 1: The questionnaire in Japanese and English: the questionnaire consisted of 21 items regarding their overall practical training and their clinical comprehension of border molding. (XLS 42 kb)

Additional file 2: The questionnaire results: The raw data used in this study. Each subject rated on a five-point Likert scale. (XLSX 22 kb)

## Abbreviations

TMDU: Tokyo Medical and Dental University
